# Post Partum Hemorrhage—Remarks upon Its Prevention and Treatment*Read before Buffalo Obstetrical Society.

**Published:** 1884-04

**Authors:** W. W. Potter


### BUFFALO

### Medical and Surgical Journal.

VOL. XXIII. APRIL, 1884. NO. 9.

### Original Communications.

### POST PARTUM HEMORRHAGE—REMARKS UPON ITS PREVENTION AND TREATMENT.*

BY W. W. POTTER, M. D.

If an apology were needed for presenting, to an assemblage of skilled medical men, a subject so time-worn and threadbare, I should find it in the fact that post partum hemorrhage possesses special and always fresh interest to the obstetric practitioner; and, in its frequent discussion he may meet with welcome suggestions which may aid him in future conflicts with this terrible calamity to the paturient woman. This is an accident of labor which differs from almost all other complications in this, that the medical attendant, whether skilled or unlearned, must make the combat with it alone and unaided by his professional brethren. While most other abnormalities of parturition permit more or less delay in treatment, thus affording time to obtain assistance, this one springs up so suddenly, oftentimes so unexpectedly, and, moreover, runs its course so rapidly, as to place the patient beyond help, if instant and efficient aid be

* Read before Buffalo Obstetrical Society.

not afforded her. And yet in these very cases, sudden and unexpected though they may be in their invasions, so appalling in their bloody physiognomy, and, withal, so quickly and certainly fatal—it is, I say, these very cases which respond with an amazing certainty to prompt and intelligent methods of treatment. In the discussion of this subject, there seems to be a great lack of definiteness and precision in regard to the conditions occasioning post partum hemorrhage, and, as a consequence, much looseness of opinion as to the real, as well as the relative, value of remedies advocated for its relief. This want of sufficient precision and definiteness has led writers to advocate, or condemn, particular methods or remedies without regard to the antecedent causes of the hemorrhage, thus leading them to urge or oppose, at times, a remedy of great value in its proper place, but useless or dangerous when otherwise employed. In order to give clearness to my own views of treatment it will be necessary, first, for me to notice briefly the causes of this direful malady.

Post partum hemorrhage may be defined as a hemorrhage from any of the parturient structures, from the moment the child is expelled until the organs have returned as nearly as may be to the condition they possessed prior to fecundation; and may be sub-divided into (*a*) primary, (*b*) secondary.

CAUSES.

I. *Uterine atony, or inertia.* Undoubtedly the most frequent cause of post partum hemorrhage, is a failure of the uterine muscle to assume that condition of firm contraction which is necessary to obliterate the blood-vessels ramifying through the walls of the organ, and thus to render them incapable of carrying blood to the torn utero-placental orifices.

II. Partial adhesion of the placenta to the uterine wall, which prevents, or renders impossible, the necessary degree of shrinkage of the organ to the complete obliteration of the uterine blood-vessels. This is, oftentimes, a condition the reverse of inertia—

the uterus being in a condition of firm contraction, but the adherent placental mass being of adequate size to prevent sufficient shrinkage of the muscular fibers of the uterus to ensure closure of the orifices of the blood-vessels. This might be included under the general head of "mechanical interference preventing the action of a uterus willing to contract;" and to this class of causes might properly be added those cases where the hemorrhage is caused by the presence of fibroids in the uterine walls, or of a polypus in its cavity.

III. *Solutions of Continuity.* Under this head I would group the various lacerations possible during labor; laceration of the uterine body, of the cervix, and of the os, as well as laceration of the vagina and perineum; and here, also, might be included hemorrhage caused by a ruptured pudendal hematocele, or vulvar thrombus; a solution of continuity may also exist in the form of cancer of the uterus, but as cancer may act in several ways as a cause of post partum hemorrhage, I shall further on place cancerous disease in a class, *per se.*

IV. A fourth class of causes embraces those where the essential shrinkage of the uterus is prevented by external abnormal peritoneal adhesions, or by a distended bladder.

V. Causes which occasion a sudden congestion of the uterine body, *e. g.*, improper exertion after labor, or powerful emotions, which latter may properly be classed as psychical causes.

VI. In a sixth class are placed those cases due to diseases ot the uterus producing tissue metamorphosis, as cancer, which, as I have before remarked, may act in various ways as an etiological factor in post partum hemorrhage; 1st, by its size it may prevent the essential shrinkage; or, 2d, it may so alter the character of the uterine tissue as to render it incapable of contraction; or, 3d, it may greatly increase the vascular supplies to the womb.

VII. Finally, a predisposing cause of post partum hemorrhage is, not seldom, a hemorrhagic diathesis.

The etiology of post partum hemorrhage might be further subdivided, perhaps, but as it is not the design of this paper to enter into a full discussion of the causes of the malady—merely to refer to them sufficiently to elucidate my own convictions of treatment—I pass at once to the consideration of the essential branch of the subject, viz., its therapeutical management.

As we have already seen, the chiefest factor in the production of flooding after labor is uterine inertia. How shall it be prevented, or, having occurred, how shall it be overcome? are questions of the greatest import.

How, then, shall uterine inertia be prevented? Assuredly the preventive treatment must be based upon a most definite conception of all the predisposing and exciting causes that can possibly occasion the accident. Dr. Thomas says that "uterine inertia is, in a large number of cases, due to mismanagement on the part of the practitioner," meaning that the attendant has not paid sufficient attention to the securing of firm uterine contraction. For myself, I should say that a principal cause of uterine laxity, and, as a consequence, of flooding after labor, is faulty management of the delivery of the placenta and membranes.

Obstetricians may be classed somewhat loosely into two parties, namely: those who regard the expulsion of the secundines of such prime importance that they do not intermit their care of the womb until this process has been entirely completed; and those who leave all or nearly all to nature, within certain limits of time, unless some emergency occurs calling for more prompt assistance.

But it is to the particular method of effecting delivery of the placental body and its attached membranes that I would invite your special attention at this time, for it is here that, within a comparatively short time, a great change has been made in practice, and which is, as I think, a great advance in the obstetric art. When I was taught midwifery, I was told to wait, after the birth of the child, some fifteen or twenty minutes, and then, if the placenta was not found lying in the vagina, uterine contraction was to be re-excited, by friction, cold or warmth, slight traction at the cord, and compression of the uterus. Failing in these measures at the end of an hour the hand was to be passed into the uterus and the placenta extracted. The theory of all teaching at that time was, that in the majority of cases the uterus spontaneously expels the placenta into the vagina, and for this we were told to wait ten to thirty minutes or longer. Now-a-days a teacher who would instruct his pupils to wait for the spontaneous expulsion of the placenta would be something of a curiosity, I imagine. Since 1853, when Professor Credé of Leipsic first made known his method of placental expression, a radical change has been wrought in the management of this stage of labor, and the doctrine of extrusion of the placenta by some form of supra-pubic pressure is now almost universally taught. Credé's method is simple in principle and easy of execution. Let me quote his own words in description of it, since we are all students as well as practitioners. Says Credé: "Its object being to reinforce the uterine contractions the accoucheur should act during a pain, and not in the interval; success is more rapid, the sooner after the expulsion of the fœtus this effort is made; nevertheless, it may succeed a quarter of an hour or even half an hour after, but delay presents an unfavorable condition.

"When the retraction of the uterus has attained its maximum in the first contraction which normally occurs after the escape of the infant, embrace the fundus and the superior part of the anterior wall of the uterus with the entire right hand placed transversely. Then press downwards and backwards, assisting if necessary with the left hand. Under this pressure the placenta and membranes are detached, then engorge the uterine orifice, sometimes even escape suddenly from the vagina, just as a cherry stone escapes when the cherry is pressed between the finger and the thumb."

In this connection I may be pardoned for giving my own method of conducting placental delivery, since a considerable experience has convinced me that the plan of maintaining the contraction of the uterus until after the birth of the placenta, by well-directed supra-pubic pressure is not a mere theory which has no value at the bedside, but one that, when properly carried out, seems to reduce the liability to post partum hemorrhage to a simple minimum. Immediately prior to the termination of the second stage of labor, I grasp firmly with the disengaged hand (usually the left) the fundus uteri and maintain moderate pressure, following the descent of the child until it clears the lower outlet. When this event happens, I direct the nurse to gently grasp the descending womb and maintain moderately firm pressure until separation of the child. This being quickly accomplished, speaking generally, I turn my attention once more to the womb, grasping the fundus with the left hand, straighten out the cord, without traction with the right, two fingers in the vagina being usually sufficient, when, in the great majority of cases, the placenta is found either partially or completely expelled from the uterus, and all that remains to do is to remove it, being careful, of course, to deliver the secundines entire, at the same time. I still maintain supra-pubic pressure upon the now contracted and hardened uterine body until the binder is applied, which I am careful to adjust low down around the hips, and with as little disturbance to the patient as possible. All debris having been removed and the bed-clothing carefully adjusted according to the season or temperature of the room, I direct the patient to remain perfectly quiet in the recumbent posture for an hour or more, at the end of which time, my offices being ended, I take my leave, provided always, that no unusual circumstance has occurred to prevent.

At the risk of being wearisome I have been diffuse in my description, for I regard as necessary to success in this plan, as, indeed, is the case in almost everything in life, that there shall be the minutest attention to details. Permit me, therefore, to further elaborate one or two important points essentially necessary to the successful employment of this method.

I. It will be observed that, from a point of time dating just prior to the expulsion of the infant to the application of the abdominal bandage, the uterus is kept within the relentless grasp of either the accoucheur or nurse. The object of this is two-fold: If pressure be not directed at once to the uterus, the womb closes over the after-birth and prevents its speedy expulsion, whereby a proportionate amount of risk of post partum hemorrhage is involved. Should flooding arise in spite of all our precautions, we have, by the maintenance of firm pressure, the uterus more immediately under our control.

II. Even the slightest traction of the cord is not permitted. This pernicious practice cannot, in my opinion, be too severely reprehended. A moment's glance at the mechanism of the expulsion of the placenta when left to itself will serve to convince any one, it seems to me, of the error of dragging, or pulling, or even of touching the cord with a view to hasten the separation or delivery of the after-birth. The placenta may be likened unto a rounded disk or flattened cake, and to find out the natural mechanism of the expulsion of this cake, it is only necessary to watch the process as nature conducts it, that is, in cases wherein the practitioner does not try to modify it in any way.

In this way it will easily be discovered that the part of the placenta presenting at the os uteri, and subsequently at the ostium vagina, is not the fœtal or amniotic surface, but the edge of the cake folded upon itself forming a wedge-shaped mass, and is thus extruded from the vulvar orifice, its long axis corresponding to the long axis of the passage, and not transverse to it, as inversion or presentation of the fœtal surface imply.

Now suppose we attempt delivery of the after-birth by traction upon the cord, what happens? Why, the placenta is inverted, the center of the disk presents at the os uteri, its circumference becomes puckered up, or folded into a cup shape, like an inverted umbrella, requiring at least twice as much space for delivery as is needed if it passes edgewise and only longitudinally folded. Moreover, into this cup-shaped cavity, formed of the placental mass, which now becomes a plug filling up the lower segment of the uterine cavity, flows the blood to fill up the partial vacuum which is thus produced. Nay, more; traction on this plug is exactly like traction on the piston of a pump. If hemorrhage does not naturally take place to fill up the void which tends to be formed beyond the placenta, then it is powerfully attracted and induced by the piston-like action of the placenta pulled by the cord. The interior of the uterus, already scarified by the separation of the placenta, requires but this pulling at the cord to be effectively cupped. But, on the other hand, suppose delivery of the after-birth by supra-pubic pressure be undertaken, what are the phenomena? Why, the placenta, instead of presenting broadside comes edgewise, its uterine surface glides along the surface of the uterus; its foldings, parallel to the length of the maternal passages, are well squeezed together, and little space is offered for the reception of blood flowing from the uterine sinuses. The uterine walls keep close to the folded placenta; the uterus contracts, forces the placenta downwards, and at last its body becomes nearly globular and empty—there is, in short, no hemorrhage worthy the name.

Let me concisely formulate the salient features and advantages of supra-pubic expression of the placenta:

I. We effect the speedy and safe removal of the placenta and so place the uterus out of its chiefest danger of hemorrhage; for when the uterus continues its contractions after the birth of the child, so as to throw off the placenta in a short period, the chance of post partum hemorrhage is, confessedly, very slight.

II. Should post partum hemorrhage, however, finally set in, we have the uterus more immediately under our control. Hemorrhage usually occurs during complete or partial separation of the placenta, and while that viscus is still within the uterus; and when it does supervene, it is generally in proportion to the non-contraction of the womb, and to the time during which the placenta remains unexpelled.

III. To assist the uterus to maintain its contractions for a short period after the birth of the child, in order to procure the separation and expulsion of the placenta, is assuredly a rational method of diminishing the risk of hemorrhage; while, at the same time, it is the surest way to secure the permanent contraction of the uterus, and thereby conduce to a speedy recovery.

IV. A partially adherent placenta may oftentimes be detached by supra-pubic compression, thus reducing the necessity for the introduction of the hand into the uterus for the purpose of accomplishing its separation, to narrower limits—a consideration of no trifling value. To avoid misapprehension upon this point, however, let me add, that if we do not succeed promptly in effecting separation by this method, then no time must be lost, if there be hemorrhage from a partially adherent placenta, in proceeding to invade the cavity with the hand and extract it.

V. A fifth advantage accruing from this plan is, undoubtedly, a diminution of cases of retention of the placenta from the so-called hour-glass contraction of the uterus.

Since the principal purpose of this paper is to consider the prophylaxis of post partum hemorrhage, I might with propriety leave the subject right here. But there are some other points in the therapeutical management of these accidents to which I desire to call attention, and with your permission, Mr. President, I will proceed to notice them now.

Let me suppose a case of hemorrhage occurring after the complete evacuation of the uterus, where the organ refuses to contract and close the orifice of the torn and bleeding utero-placental vessels; where in spite of supra-pubic compression, friction, ergot, the use of nervous and cerebral stimuli, cold in various ways, etc.; in short, where all the milder expedients, well known to us all, have failed to produce sufficient shrinkage of the uterine muscle to obliterate the blood-vessels and thus *safely* stop the flow.

I present a case where the hemorrhage has failed to be controlled by the use of any or all the usual and milder expedients; where the symptoms are most urgent and where we realize that if the flow continues much longer, a fatal termination is inevitable. It cannot be denied that now and then such a case is met with in spite of the most completely organized prophylactic method. It may be the result of a prolonged and exhausting labor, when the uterus seems to have expended all its contractile power in expelling the infant; it may occur in a pale, weakly, anæmic female, with an enervated constitution, already exhausted by previous disease; or it may result from a hemorrhagic diathesis. Whatsoever the primary cause of this direful condition of things may be, it is due now to a suspension of the excito-motor function, and this sluggish, this inert, this paralyzed uterus, so to speak, must be awakened to activity by some powerful stimulant, and that instantly, or the patient will die.

Under these conditions what must be done? Many are the methods suggested, but the all-important question is, which is the best one for the case unto which we may be ministering.

Dr. Barnes says inject the perchloride of iron; and, no doubt, under his skilful hand it is a potent and useful remedy. If the blood be still running, it is instantly seized at the mouths of the vessels, which become sealed up with coagula. It also constringes the inner surface of the uterus, and thus further closes the vessels. The system then has time and opportunity to rally, and by and by the contractile power returns.

But, is it a remedy entirely devoid of danger, *per se*? If we analyze the testimony of the men who have the greatest experience in its use I fear we shall arrive at the conclusion that, in not a few instances, has the injection itself caused death; and even Dr. Barnes, himself the first to propose, and the strongest advocate of the remedy, admits its danger under certain conditions. To quote his words, in which he clinches a powerful argument for its use: "If occasionally death follows and is apparently accelerated by the iron injection, we have, on the other hand, to remember that it was used as a last resource, when the patient was likely to die even if nothing were done, and that even under these unpromising conditions *many lives, to all appearances doomed, have been saved.*" These words, from the pen of one of the most distinguished obstetricians in Great

Britain, must carry great weight, and particularly when we reflect that he is a man who always has "a reason for the faith that is in him."

Dr. Lombe Atthill, the distinguished ex-president of the Obstetrical Society of Dublin, has thrown the weight of his powerful influence in favor of the use of styptic injection. In relating a case which he lost from post partum hemorrhage, and whose bedside he never left from the termination of the first stage of labor until her death, he says: "Moreover, I regret to say, I did not inject the perchloride of iron. It was the first case of severe hemorrhage which occurred in my practice after the method had been brought under my notice by Dr. Barnes; and, like many of my brethren now-a-days, I feared to use this, to me, new and powerful remedy. I now firmly believe that to this timidity the death of my patient—a young wife, and a young mother—was due. I feel that she might still be alive if only I had used a remedy I knew of, but had not the courage to employ. This, however, I have to compensate me, that though since then I have stood beside the bed of more than one whose life seemed to me in greater peril than hers, to whom I have just alluded, no such scene as that I then witnessed followed, nor do I believe it ever will again. The lesson I that day learned taught me the utter inutility of ordinary means at our command for the arrest of post partum hemorrhage in a certain class of cases."

He then relates, in detail, several cases where he successfully injected the iron styptic, and concludes as follows:

"For myself, I have arrived at the following conclusions:

I. "That cases of post partum hemorrhage occur in which the injection of the perchloride of iron, or some similar styptic, is alone capable of arresting the hemorrhage.

II. "That the injection of such styptic does not necessarily increase the tendency which exists in such cases to the occurrence of pyæmia, septicæmia, or peritonitis.

III. "That the treatment is especially applicable to anæmic patients, and those of a hemorrhagic diathesis.

IV. "That while it should never be had recourse to unnecessarily, it should not, on the other hand, be delayed too long."

Other obstetricians, of prominence in that country, take ground in opposition to this method, the chiefest of whom is Dr. Snow Beck; while on this side of the water it has its advocates and its opponents, as well. My own experience with the remedy is limited to a single case, in which it succeeded admirably; yet, I confess, I should hesitate, in view of all that has been said of its deleterious effects, to inject it again except in the direst necessity. I should be disposed, instead, to swab out the uterus with a sponge saturated with the styptic.

The injection of the tincture of iodine has been used with much success in post partum hemorrhage, and its advocates say that in comparison with the iron it has the advantage of being perfectly safe. Besides this the antiseptic influence of the iodine upon the uterine and vaginal mucus membrane is well known. As an excito-motor agent iodine is probably, at least, equally as good as the iron, while incapable of causing thrombi in the uterine vessels. In view of these facts one would feel justified in resorting to the iodine earlier than to the iron, and in this respect also an advantage may be gained for the patient, since the use of the iron is expressly limited to cases deemed hopeless under ordinary management.

Another most effectual method of controlling post partum hemorrhage is by the injection of hot water into the uterine cavity. The superiority of hot to cold water, as a hemastatic in surgery, has come to be pretty well understood by the profession in general. All surgeons who use hot water when making operations agree that it is vastly to be preferred to cold, and that nothing could persuade them to go back to the old way. There is no class of operations which have so rapidly advanced, in point of safety, as abdominal sections, and one of the chief precautions acknowledged to be desirable in that class is that the peritoneum shall be kept warm. Fancy an ovariotomist to-day sponging out the abdomen with cold water during the operation! Such a spectacle would cause a universal shudder of horror from an insulted and outraged profession. It has been suggested that, when used against post partum hemorrhage, hot water acts on nerve centres situated in the substance of the uterus itself. This action may be direct, or it may be reflex, or it may be both. It certainly is a remedy which possesses the advantage of being easily attainable at all times, and so far as we can judge, it also possesses the invaluable merit of being absolutely safe.

Dr. Penrose of Philadelphia, a most accomplished obstetrician, has somewhat recently brought to the notice of the profession a remedy, which, if it is capable of accomplishing all he claims for it, should take a place in the front rank, if, indeed, it should not supplant all the others, in the treatment of post partum hemorrhage when not controlled by ordinary methods. This remedy is common vinegar, and his method of using it may best be described in his own words. He says: "I pour a few tablespoonfuls into a vessel, dip into it some clean rag or a clean pocket handkerchief. I then carry the saturated rag with my hand into the cavity of the uterus and squeeze it; the effect of the vinegar flowing over the sides of the cavity of the uterus and through the vagina is magical. The relaxed and flabby uterine muscle instantly responds. The organ at once assumes what I will term its gizzard-like feel, shrinking down upon and compressing the operating hand, and in the vast majority of cases all hemorrhage ceases instantly; should one application of vinegar fail to secure sufficient contraction, the hand can be withdrawn, and a second or even a third application can be made, until the uterus shall contract sufficiently to stop the flow of blood."

He claims the following advantages for the remedy:

I. It can always be easily obtained—every household, even the humblest, having a vinegar cruet.

II. It can be applied instantly and without apparatus.

III. It always cures the hemorrhage, or rather, I should say, it has never failed to do so in his practice. It is sufficiently irritating to excite the most benumbed and sluggish uterus to contract, while, at the same time, it is not so irritating that its subsequent effects are injurious.

IV. It is an admirable antiseptic. Professor Zweifel of Erlangen, in a paper published not long ago on the prevention of puerperal fever, considers vinegar an excellent substitute for carbolic or salicylic salts.

V. It acts on the lining membrane of the uterus, and on the gaping orifices of the torn utero-placental vessels as a valuable astringent.

But, gentlemen, I am admonished of the flight of time, and that I have already exhausted your patience, though, by no means, have I exhausted this interesting subject. While, therefore, it would have been satisfactory to have discussed the treatment, not merely of hemorrhage when caused by uterine inertia, but when due to some of the other causes to which I referred at the outset, I must yet forego the pleasure it would have afforded me to do so.

I should be glad, also, to make some reference to other remedies employed in the treatment of post partum hemorrhage—notably among them the hypodermic use of ergotine, galvanism, and the subcutaneous injection of sulphuric ether, as recently recommended by Macan of Dublin—but I cannot consent to further trespass upon your valuable time.

Let me, in concluding this paper, formulate some of the doctrines, which it has sought to enforce.

My first proposition is—

That post partum hemorrhage can, in a large majority of cases, be prevented by a proper management of the third stage of labor. After labor the obstetrician should force the uterus into a condition of firm contraction, and should not consider the third stage of labor as completed with the delivery of the placenta. The expulsion of the placenta is not the third stage of labor, although it usually occurs during the third stage. It is an epiphenomenon of this stage. The third stage of labor may be defined as a tonic contraction of the uterus; in this stage there can be no hemorrhage, and to secure it is the object of all methods proposed for the relief of post partum hemorrhage.

My second proposition is—

That when the excito-motor function is suspended; when neither by peripheral excitation, nor by centric stimulus, the nerve force can be drawn or sent from the spinal cord to the uterus in sufficient intensity to cause contraction of the uterine muscle, and thus obliterate the torn orifice of the utero-placental vessels, then it is that intra-uterine injections or swabbings become a most precious method of rousing the sluggish, relaxed and almost paralyzed uterus into action. I should advocate their use in the following order:

1. The injection of hot water.
2. The swabbing of the uterine cavity with vinegar.
3. The injection of iodine.
4. The swabbing of the cavity with the iron styptic, or, finally, the injecting of it into the uterus, since there are undoubtedly cases in which the latter will alone arrest the hemorrhage—cases in which I should stand in much greater fear of the bleeding than the remedy.

Finally, and as the sum of all, permit me to offer the suggestion that we should never attend a case of midwifery unprepared to meet the possibility of post partum hemorrhage.

The careful practitioner will always go armed with ergot, opium, ammonia, a good syringe, and some form of styptic or iodine; for, should the emergency arise, he will have no time to send for them. These form an essential part of the armamentarium of the obstetrician, to be supplemented with patience, firmness, a clear head, a kind heart, and above and beyond all, that inestimable element, contributing alike to the success of soldier and physician—*courage.*

